# Oxyfunctionalization of Benzylic C-H Bonds of Toluene Mediated by Covalently Anchored Co-Schiff Bases

**DOI:** 10.3390/molecules27165302

**Published:** 2022-08-19

**Authors:** Guojun Shi, Yuxin Liang, Hongyu Zhou, Zhengliang Zhao, Wenjie Yang

**Affiliations:** School of Chemistry and Chemical Engineering, Yangzhou University, Yangzhou 225002, China

**Keywords:** toluene, oxyfunctionalization, Co-Schiff base, covalent anchoring, electronic effect

## Abstract

Oxyfunctionalization of toluene to value-added benzaldehyde, benzyl alcohol and benzoic acid is of great significance. In this work, Co-Schiff bases were immobilized on commercial silica gel by covalent anchoring, and resulting catalysts were used to catalyze the oxidation of toluene in the presence of the cocatalyst N-hydroxyphthalimide (NHPI). The catalysts exhibited excellent textural and structural properties, reliable bonding and a predomination of the cobaltous ions. The catalyst synthesized by diethylamino salicylaldehyde (EASA) possessed a grafting density of 0.14 mmol/g and exhibited a toluene conversion of 37.5%, with predominant selectivities to benzaldehyde, benzyl alcohol and benzoic acid under solvent-free conditions. It is concluded that the effect of ligands on their catalytic performance might be related to their electron-donating or -withdrawing properties.

## 1. Introduction

Oxyfunctionalization of benzylic C-H bonds of toluene can produce value-added oxygen-containing compounds such as benzyl alcohol, benzaldehyde and benzoic acid, which are endowed with important applications in perfumes, pesticides, dyes, pharmaceuticals, preserves, etc. [1,2]. A direct oxidation of the primary C-H bonds of toluene can avoid prefunctionalization of substrates and thus simplify a synthetic route [3]. Furthermore, a successful transformation from toluene to its derivatives will allow the oxyfunctionalization of a library of compounds containing benzylic C-H bond(s) [4]. Benzyl alcohol, benzaldehyde and benzoic acid can be prepared via a chlorination of toluene followed with a sequent hydrolysis [5]. Such a production method, however, will lead to severe corrosion of equipment and chlorination contamination of the resulting products, which will exclude their applications from foods, cosmetics and pharmaceuticals [6,7].

Molecular oxygen has received increasing attention in selective oxidation of hydrocarbons as a terminal oxidant due to its characteristics in cost, environmental friendliness and availability [8,9]. However, poor reactivity is observed under mild reaction conditions when alkyl-substituted aromatics are used as the substrates because of a spin-flip restriction between the ground triplet molecular oxygen and the sp^3^ hybridized C-H bonds [10,11]. More severe reaction conditions are employed to promote the conversion of the substrates, which leads to a terribly low selectivity of the desired O-containing organics and a lot of byproducts with poor value [12,13]. In this regard, a carefully fabricated catalytic system is required to realize a highly efficient transformation from toluene to its O-containing derivatives with an enhanced value under relatively mild reaction conditions.

Vapor-phase oxidation of toluene to the desired organics exhibits a high space-hour yield and an enhanced efficiency under a relatively high temperature, but the reactivity of the substrate has to be limited to avoid a noticeable overoxidation [14]. The production of CO_x_ and other small molecules can be efficiently inhibited when toluene oxidation proceeds in the liquid phase [15,16,17].

The combination of cobalt acetate and NaBr was found active for the oxyfunctionalization of toluene in acetic acid as a solvent [18]. Ishii and his collaborators found that hydroxyphthalimide (NHPI) and Co(OAc)_2_ (NHPI/Co) can remarkably promote the transformation from toluene to benzoic acid with a conversion high up to 84% at ambient temperature and oxygen [12,19]. The transformation realized a high toluene reactivity under normal temperature and pressure but suffered from severe corrosion and contamination. Gold-palladium nanoparticles were fabricated to catalyze the oxidation of the primary C-H bonds of toluene under mild solvent-free conditions, and a high selectivity to benzyl benzoate was observed [14]. Hexafluoropropan-2-ol (HFIP) was found to have a remarkable promotional role in the transformation from toluene to benzaldehyde, which might be ascribed to its stabilizing capability to radical intermediates and inhibiting role on the further oxidation of benzaldehyde to benzoic acid [20,21,22]. However, the application prospect of the transformation is eclipsed by some difficulties, such as the separation of the homogeneous NHPI/Co catalysts and the usage of the expensive solvent HFIP, which possesses a low boiling point and will be inevitably depleted. In this regard, a transformation of toluene on immobilized catalyst(s) under solvent-free conditions may open access to the industrial application of the transformation.

Cobalt catalysts show versatile capability in isomerization, carbonylation, alkylation, peroxidation, etc. [23,24,25,26]. Comparatively, immobilized Co catalysts exhibit superior performances in separation, recycling and reusability to the homogeneous ones and thus a more promising application prospect. Silica [27], alumina [28], chitosan [29] and mesoporous carbon [30] were used to load or anchor Co catalysts, and the resulting catalysts were used for the oxyfunctionalization of toluene. In our previous work, impregnation [16] and coprecipitation [31] were used to prepare immobilized Co catalysts, and the prepared CoOx/SiO_2_ catalysts were tested for liquid phase oxidation of toluene. A high toluene conversion and a high selectivity to benzaldehyde were observed in dioxygen when HFIP was used as the solvent [16]. A low reusability, however, was observed.

In this work, 3-aminopropyltriethoxysilane (APTES) was used as a linking molecule to covalently anchor the Co-Schiff base onto a commercial SiO_2_ support, and the resulting immobilized Co-Schiff base catalysts were used to catalyze the oxofunctionalization of toluene in the presence of NHPI under solvent-free conditions. Furthermore, the reusability and structure–activity relation of the catalysts are investigated, and a possible mechanism is proposed.

## 2. Results and Discussion

### 2.1. Structural and Textural Properties

#### 2.1.1. Composition of Catalysts

The fresh Co-Schiff base catalysts were analyzed by an elemental analyzer and ICP-OES to determine their compositions and compared with that of the recycled one from the sixth catalytic test (SiO_2_-APTES-EASA-Co-R6), and the results are shown in Table 1. The fresh catalysts showed a concentration of nitrogen element ranging from 0.84 to 1.43 mmol/g, and a concentration of Co element from 0.12 to 0.17 mmol/g. There was little difference in the concentration of the cobalt element for different fresh catalysts, and the discrepancy in the concentration of the N element can be ascribed to the various grafting densities of ligands. The recycled catalyst presented lower concentrations of N and C elements, which can be related to a possible elution under reaction conditions.

**Scheme 1:**
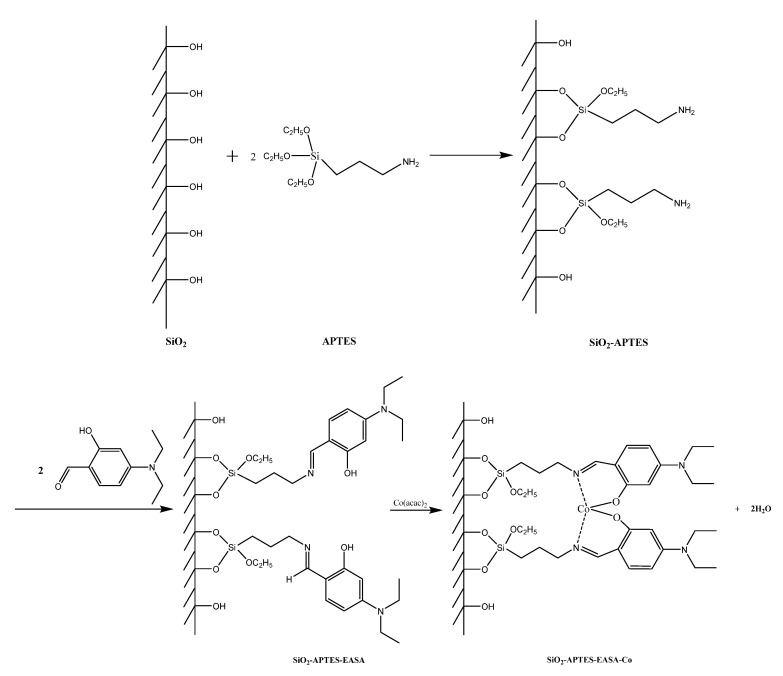
Synthetic route of the covalently anchored Co-Schiff base catalyst SiO_2_-APTES-EASA-Co.

It is worthwhile to note the differences in C/N ratio and Co/N ratios compared with their theoretical values. For example, the atomic ratio of C/N of the catalyst SiO_2_-APTES-EASA-Co (8.9) was higher than its theoretical value calculated according to Scheme 1, which can be ascribed to the adsorption of EASA with a C/N ratio of 11, meaning that some EASA may adsorb on the support without forming the SiO_2_-APTES-EASA complex. However, a lower Co/N ratio (0.17) in comparison with the theoretical value (0.25) might arise from some isolated SiO_2_-APTES groups that were not anchored with EASA, and/or some isolated SiO_2_-APTES-EASA that were not exchanged for cobaltous ions with Co(acac)_2_ to form a Co-Schiff base due to a further distance between ligands. The catalyst recycled from the sixth catalytic test (SiO_2_-APTES-EASA-Co-R6) showed decreased elemental contents, which can be attributed to the elution of some precursors which are physically adsorbed on the support under reaction conditions.

#### 2.1.2. Textural Properties

The support, silica gel (SiO_2_), exhibited excellent textural properties with a specific surface area of 418 m^2^/g, a pore volume of 0.62 mL/g and an average pore diameter of 10.9 nm, providing a feasibility for its surface functionalization and an availability for anchored active sites (Table 2). An apparent decrease in the textural properties of the support was observed after they were decorated by surface amination and covalent anchoring by Schiff base ligands followed by an exchange of cobaltous ions. The functionalization in the inner surface of silica gel will cause a decrease in the pore diameter of silica gel or partial blockage, and thus some surface of the support is not accessible for N_2_ molecules under the temperature of liquid nitrogen. This may suggest a successful surface functionalization of the support, agreeing with the results acquired by elemental analysis shown in Table 1. The catalyst (SiO_2_-APTES-EASA-Co) showed a slightly low specific surface area in comparison with the other ones, which might be related to a relatively low grafting density. The spent catalyst (SiO_2_-APTES-EASA-Co-R6) exhibited a slight increase in specific surface area, pore volume and average diameter in comparison with its fresh counterpart, probably due to a removal of the species physically adsorbed.

#### 2.1.3. Morphology and Element Distribution

**Figure 1:**
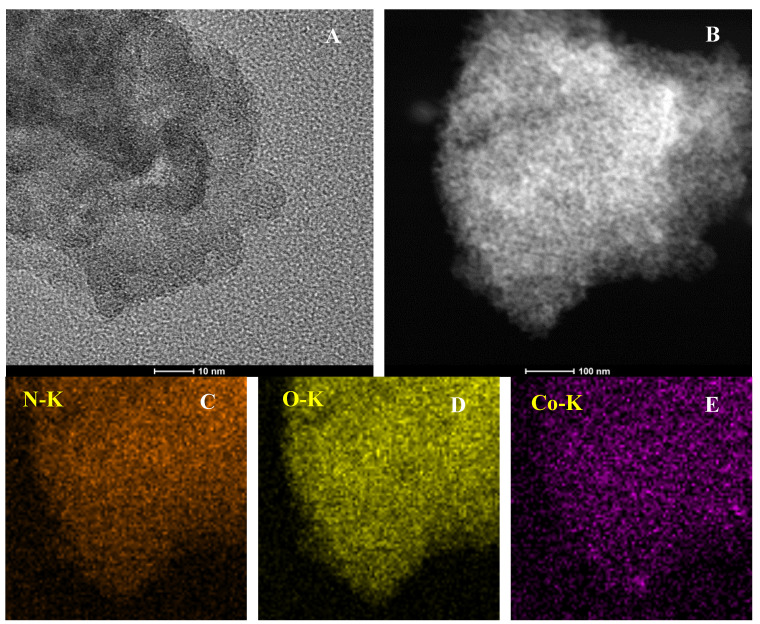
HRTEM (**A**) and STEM (**B**) images of the covalently anchored catalyst SiO_2_-APTES-EASA-Co and its elemental mapping images (**C**–**E**).

The covalently anchored Co-Schiff base catalysts were analyzed by a transmission electron microscope (TEM) and the images are shown in Figure S1. There were no particles observed by TEM, indicating an absence of apparent agglomeration and serious decomposition of the Co precursor. Furthermore, the covalently anchored Co-Schiff base catalyst SiO_2_-APTES-EASA-Co was analyzed by a high-resolution transmission electron microscope (HRTEM) and its HRTEM, STEM and elemental mapping images are shown Figure 1. The HRTEM and STEM images of the catalyst showed an amorphous structure and the absence of agglomerations clearly. The elemental mapping images presented high dispersion degrees of the N, O and Co elements. The distributions of N, O and Co elements are almost the same, suggesting a successful anchoring of the Co-Schiff base on the surface of the support SiO_2_. In addition, the energy dispersive X-ray spectrum (EDS) of the covalently anchored catalyst SiO_2_-APTES-EASA-Co also showed the presence of a Co element (Figure S2), which was in line with results from the elemental analysis and mapping and suggested the successful anchoring of the Co-Schiff base.

#### 2.1.4. Structural Properties

**Figure 2:**
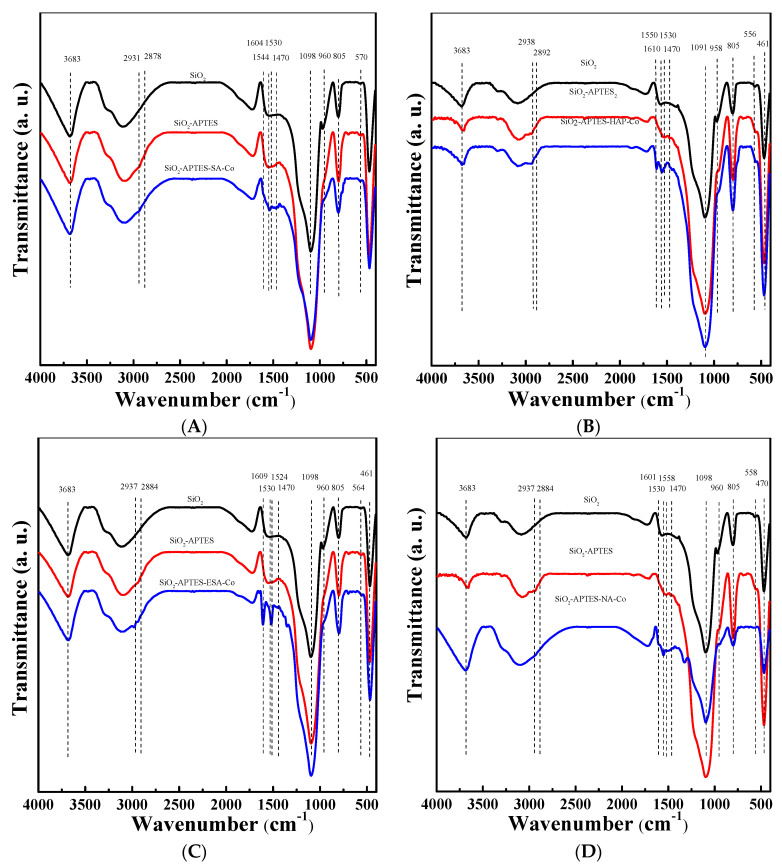
FT-IR spectra of the covalently anchored catalysts SiO_2_-APTES-SA-Co (**A**), SiO_2_-APTES-HAP-Co (**B**), SiO_2_-APTES-EASA-Co (**C**) and SiO_2_-APTES-NSA-Co (**D**) in comparison with the support SiO_2_ and the intermediate SiO_2_-APTES.

The anchored Co-Schiff base catalysts were characterized by FT-IR and the obtained spectra were compared with those of the support SiO_2_ and the aminated intermediate (Figure 2). The support SiO_2_ presents IR absorption at 3683 cm^−1^ and 960 cm^−1^, which can be partially attributed to surface silanol groups. This provides a possibility for a combination with the linker APTES via covalent bonds [32,33]. The aminated SiO_2_ (SiO_2_-APTES) exhibited asymmetric and symmetric stretching vibration modes of saturated C-H bonds at 2931 cm^−1^ and 2878 cm^−1^ and their bending mode at 1470 cm^−1^. Furthermore, the band at 1530 cm^−1^ can be assigned to the bending vibration of N–H bonds. Therefore, it is reasonable to conclude that the support SiO_2_ was successfully decorated by the linker APTES by covalent bonds. For the anchored Co-Schiff base catalysts, the characteristic absorptions of C=N bonds at 1600–1610 cm^−1^ and Co–O bonds at 556–570 cm^−1^ well confirmed the immobilization of Co-Schiff bases.

**Figure 3:**
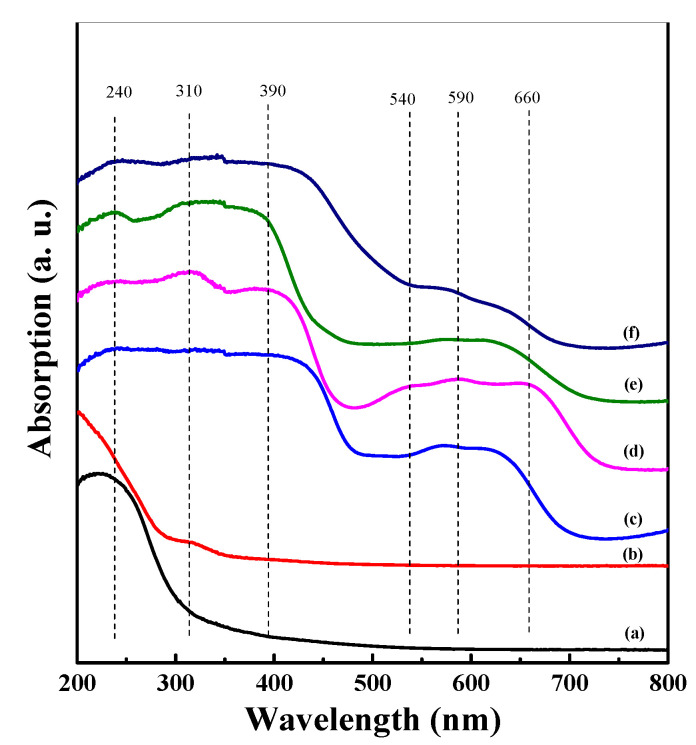
UV-Vis spectra of the support SiO_2_ (a), the intermediate SiO_2_-APTES (b) and the covalently anchored catalysts SiO_2_-APTES-SA-Co (c), SiO_2_-APTES-HAP-Co (d), SiO_2_-APTES-EASA-Co (e) and SiO_2_-APTES-NSA-Co (f).

The covalently anchored Co-Schiff base catalysts were analyzed with a UV-Vis DR spectroscope and compared with the supports before and after amination (Figure 3). The support SiO_2_ and its aminated counterpart presented a strong absorption edge at 240 nm, which can be attributed to the absorption of SiO_2_ cores [34]. The anchored Co-Schiff base catalysts present absorptions at ca. 240 nm and 310 nm, which can be assigned to the π-π* and n-π transitions due to the presence of phenyl rings and C=N bonds, respectively [35]. This involves the Schiff base ligand, suggesting a successful covalent anchoring on the support. The absorption at ca. 390 nm of the Co-Schiff base catalysts can be related to the metal–ligand charge transfer of the d-π* transitions [36,37], and the absorptions between 500–700 nm might arise from d–d transitions of cobaltous atoms [38]. In the light of this, the covalently anchored Co-Schiff base catalysts were believed to have successful synthesis via the ligands SA, HAP, EASA and NSA.

**Figure 4:**
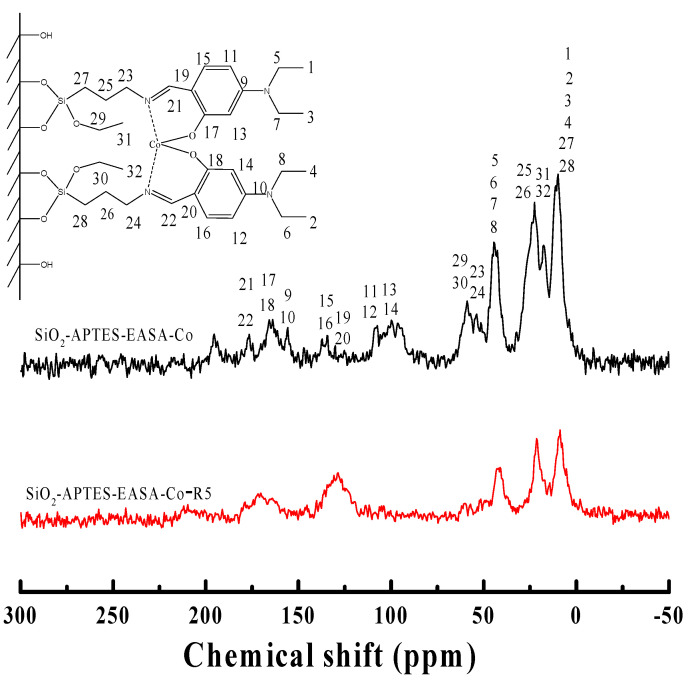
The ^13^C NMR spectra of the fresh catalyst SiO_2_-APTES-EASA-Co and the catalyst SiO_2_-APTES-EASA-Co retrieved from the 6th catalytic test.

The synthesized SiO_2_-APTES-EASA-Co catalyst was analyzed by a ^13^C NMR spectrometer to identify the anchored organic moiety (Figure 4 and Table S1). The C1–C22 and C23–C32 in the ^13^C NMR spectrum of the fresh SiO_2_-APTES-EASA-Co catalyst can be well assigned to the ligand EASA and the linker molecule APTES, respectively. This indicated a successful anchoring of the organic moiety and agreed with the results from the elemental analysis (Table 1), HRTEM and elemental mapping images (Figure 1), FT-IR and UV-Vis spectra (Figure 2 and Figure 3). Furthermore, the catalyst recycled from the sixth catalytic test exhibited a ^13^C NMR spectrum similar to that of its fresh counterpart, suggesting an excellent stability under reaction conditions.

It is noted that there was an apparent decrease in the intensity of the ^13^C NMR spectrum of the spent catalyst in comparison with its fresh counterpart, and some peaks seemingly presented some shifts. The possible reasons lie in: (1) a low concentration of the grafted organic moiety led to a weak response and a poor resolution ratio for both fresh and spent catalysts, which is a challenge for a complete identification and correct comparison; (2) the changes in the spent catalyst in ^13^C NMR spectrum may be related to a possible elution of an anchored organic moiety, a contamination in the process of regeneration and/or a heterogeneity of the sample tested for NMR. In addition, the ^13^C NMR spectra of the fresh catalysts fabricated by other Schiff base ligands are shown in Figure S3–S5, and the carbon atoms of the catalysts were well assigned and indicated a successful covalent anchoring of the linking molecule and the ligands.

### 2.2. Thermal Properties

**Figure 5:**
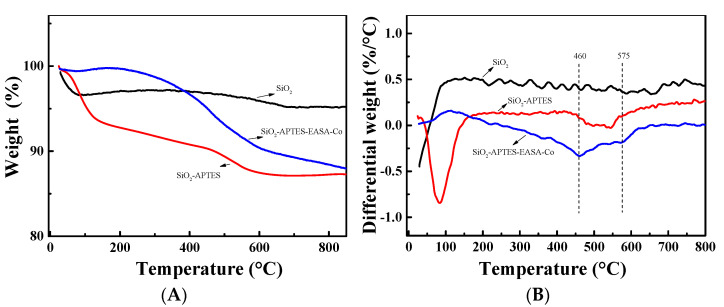
TGA (**A**) and DTG (**B**) curves of the support SiO_2_, the intermediate SiO_2_-APTES and the covalently anchored catalyst SiO_2_-APTES-EASA-Co.

The synthesized Co-Schiff base catalyst (SiO_2_-APTES-EASA-Co) was characterized by thermogravimetric analysis (TGA) at a temperature range of 30–900 °C in a nitrogen flow of 25 mL/min, and the obtained TG and DTG curves are compared with those of the supports before and after amination (Figure 5). The support SiO_2_ presented a weight loss before 120 °C due to a removal of physisorbed water, and a negligible weight loss after the temperature might arise from the deprivation of surface hydroxyl groups. Compared to the support SiO_2_, the aminated support (SiO_2_-APTES) exhibited an apparent weight loss from 120 °C to 600 °C besides the removal of physisorbed water at a lower temperature range, which can be ascribed to the decomposition of the organic moiety from the anchored APTES. It is worthwhile to note that the Co-Schiff base catalyst SiO_2_-APTES-EASA-Co presented a higher decomposition temperature than the aminated support, which can be related to the covalent anchoring of the ligand EASA with the linker APTES on the surface of SiO_2_. The weight losses around 450 °C and 575 °C might be attributed to the decompositions of the anchored Co-Schiff base and the debonding of the APTES, respectively. It is also interesting to find that the synthesized catalyst presented no appreciable weight loss before 200 °C except for the physisorbed water, indicating an excellent thermal stability for the desired catalytic reaction. Additionally, the thermogravimetric analyses of other fresh catalysts fabricated by covalent anchoring are shown in Figure S6. There were similar decomposition temperatures in comparison with the catalyst SiO_2_-PTES-EASA-Co, and the discrepancy of weight loss can be related to different grafting densities and possible adsorbate(s).

### 2.3. Chemical States of Co Element

**Figure 6:**
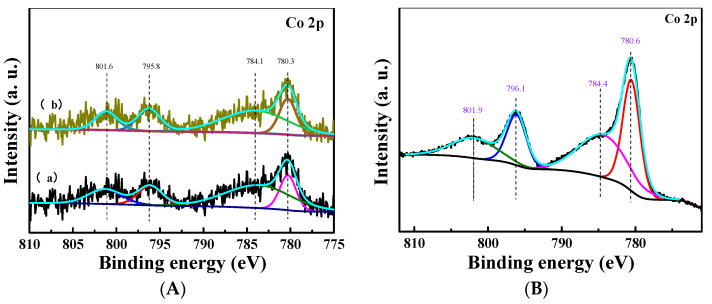
Co 2p XP spectra of the fresh (a) and the spent (b) SiO_2_-APTES-EASA-Co catalysts (**A**) in comparison with the Co 2p XP spectrum in Co(acac)_2_ (**B**).

The fresh and spent Co-Schiff base catalysts synthesized by the ligand EASA were characterized by XPS to probe the chemical states of the cobalt atoms in them and compared them with that of the precursor Co(acac)_2_ (Figure 6 and Figure S7). The survey XP spectra of both the fresh and the spent catalysts exhibited the presence of the Co, N, O and Si atoms, indicating a successful anchoring of the organic moiety Co-Schiff base and its relative stability under the applied synthesis conditions (Figure S7). The core level XP spectrum of Co in the fresh catalyst presented binding energies at 780.3 eV and 796.0 eV, accompanied by two strong satellite peaks at 784.1 eV and 801.1 eV, respectively (Figure 6A). This suggests a classic characteristic of cobaltous ions, indicating that the Co^2+^ ions have been coordinated to the Schiff base except for a possible formation of CoO particles in the process of synthesis [39,40,41,42,43,44]. Compared with the binding energy of Co^2+^ in the referenced reagent Co(acac)_2_ (Figure 6B), a decrease of 0.3 eV in the fresh catalyst was observed. It was also noted that the binding energies of the atoms in the spent catalyst were roughly in agreement with those of the fresh one, demonstrating a stability of the chemical state of Co atoms in the synthesized catalyst under reaction conditions.

### 2.4. Catalytic Performance

The covalently anchored Co-Schiff base catalysts synthesized were tested via toluene oxidation under solvent-free conditions using molecular oxygen, and the results are summarized in Table 3. It is clear that the synthesized catalysts are active for the transformation from toluene to its oxofunctionalization products, and the discrepancy in catalytic activity can be related to the ligands anchored. On the basis of the catalyst synthesized by salicylaldehyde, the catalysts synthesized by ligands carrying an electron-donating group such as methyl (HAP) or diethylamino (EASA) exhibited an enhanced catalytic activity. However, the catalyst synthesized by ligands with an electron-withdrawing group such as nitryl (NSA) presented a much lower toluene conversion. The electronic effect of the substituted groups of the ligands on catalytic activity can be related to the density of electronic clouds around Co ions. The electron-donating groups decrease the chemical state of the Co^2+^ ions, which facilitates the activation of dioxygen and leads to an enhanced activity of the synthesized catalyst for toluene oxidation in comparison with the one synthesized by the ligand salicylaldehyde without a substituted group. It is also noted that the synthesized catalyst with an electron-donating diethylamino group on the benzene ring of the ligand possesses the highest catalytic activity among the catalysts investigated, presenting a toluene conversion of 20.8%. The products from the oxyfunctionalization of toluene benzaldehyde, benzyl alcohol and benzoic acid possess an added value compared with the starting material toluene. Moreover, the absence of solvent and the use of molecular oxygen as the oxidant will endow the transformation with a more promising prospect in industrial applications [45,46]. It is also interesting to note that the homogeneous intermediate Co-EASA presented a much lower catalytic activity compared with its counterpart immobilized on SiO_2_ (SiO_2_-APTES-EASA-Co), in spite of an extended reaction duration.

Briefly, the covalently anchored Co-Schiff base catalysts, which were fabricated in this work, exhibited a relatively high catalytic activity for selective aerobic oxidation of toluene under solvent-free conditions in comparison with those reported in the literature (Table S2). The anchored catalyst with electron-donating groups (SiO_2_-APTES-HAP-Co and SiO_2_-APTES-EASA-Co) presented a slightly increased reactivity of toluene with respect to the referenced catalyst SiO_2_-APTES-SA-Co. However, it is clear that the catalyst with a strong electron-withdrawing group (SiO_2_-APTES-NSA-Co) showed much lower toluene conversion.

Different ratios of toluene/NHPI were adopted to investigate the catalytic performance of the synthesized SiO_2_-APTES-EASA-Co in toluene oxidation under solvent-free conditions when the NHPI/Co was kept constant (Table 4). It was found that an enhancement in toluene/NHPI ratios led to a decrease in the reactivity of toluene, which can be ascribed to less contact time of toluene with the NHPI/Co catalyst. In the case of a toluene/NHPI ratio of 10, the toluene conversion amounted to 37.5%, and the resulting products were predominantly benzaldehyde, benzyl alcohol and benzoic acid. It was also noticed that a higher selectivity to benzoic acid resulted when more toluene was converted at a lower toluene/NHPI ratio. This can be related to the further conversion from the product benzaldehyde to benzoic acid and from benzyl alcohol to benzaldehyde and/or benzoic acid.

Effect of reaction time on toluene conversion and the selectivities to benzaldehyde, benzyl alcohol and benzoic acid were investigated, and the results are shown in Table S3. There was an apparent increase in toluene conversion while extending the reaction time. However, the selectivities to both benzaldehyde and benzyl alcohol presented a decreasing trend with an increasing reaction duration. The decrease in the selectivity to benzaldehyde should be attributed to its further oxidation to benzoic acid, which proceed via a cleavage of aldehydic C-H bonds and an addition of molecular oxygen [2]. However, a low selectivity to benzyl alcohol might be related to its further transformation into benzaldehyde via a radical mode [47] or dehydrogenation [48].

Different substrates were tested to investigate the catalytic performance of the covalently anchored Co-Schiff base in the presence of NHPI, and the results are shown in Tables S4–S6. Cumene was selectively converted into cumyl hydroperoxide with a conversion of 37.8% and a selectivity of 73.2% under the same reaction conditions as toluene oxidation. The predominating oxidation products of ethylbenzene and paraxylene were acetophenone and p-methyl benzoic acid with conversions of 26.1% and 57.8%, respectively. This indicated the Co-Schiff base catalyst synthesized by covalent anchoring in this work is active for benzylic C-H bonds in different substrates, and a promising prospect.

In order to probe the origin of the catalytic activity of the synthesized Co-Schiff bases, the control experiments were carried out and the results are outlined in Table 5. There was no toluene conversion observed in the absence of a catalyst, nor with the synthesized Co-Schiff base catalyst. A negligible reactivity of toluene was found when only NHPI was used as the catalyst. However, a toluene conversion high up to 31.3% was observed when the combination of the SiO_2_-APTES-EASA-Co and NHPI was adopted under applied solvent-free conditions using dioxygen as the oxidant. This suggests that the observed transformation from toluene to its oxyfunctionalization products was catalyzed by the combined catalysts synergistically under adopted reaction conditions.

### 2.5. Catalytic Reusability

**Figure 7:**
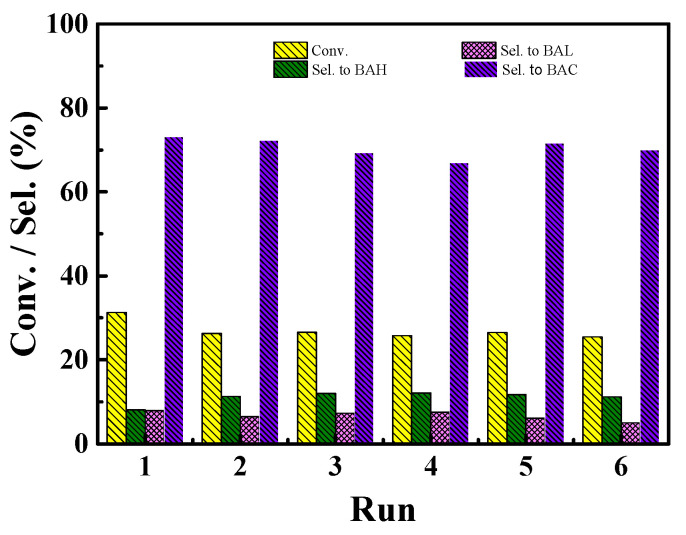
Reusability of the catalyst SiO_2_-APTES-EASA-Co for solvent-free oxidation of toluene via molecular oxygen. Toluene/NHPI (40), NHPI/Co (80), 2.6 MPa, 100 °C, 1 h. BAH: benzaldehyde, BAL: benzyl alcohol, BAC: benzoic acid, DBE: dibenzyl ether.

The catalytic reusability of the anchored catalyst SiO_2_-APTES-EASA-Co was probed under the same reaction conditions as those applied for the fresh catalysts, and the results are presented in Figure 7. A slightly lower toluene conversion was observed when the catalyst recycled from the first catalytic test was evaluated, which can be attributed to the elution of the catalytically active components physically adsorbed on the surface of the support and the possible carbonaceous deposit generated under reaction conditions. It is worthwhile to note that the toluene conversions and the selectivities to its oxyfunctionalization products were kept relatively stable from the second to sixth runs. The results indicated an excellent catalytically reusable capability, which is vital for its prospect in industrial applications. Furthermore, the excellent reusability of the anchored catalyst is well in agreement with its stability in elemental composition (Table 1), textural (Table 2), structural (Figure 4) and thermal properties (Figure 5), and surface chemical state (Figure 6).

## 3. Experimental Section

### 3.1. Synthesis of Immobilized Co-Schiff Base Catalysts

#### 3.1.1. Preparation of Surface-Aminated SiO_2_

Dried silica gel (SiO_2_, 6.4 g, S_BET_: 418 m^2^/g, average pore diameter: 10.9 nm, Sinopharm, Beijing, China) and (3-aminopropyl) triethoxysilane (APTES, 20 mmol, Sinopharm) were added to dichloromethane (CH_2_Cl_2_, 100 mL, Sinopharm). The surface amination of the support silica gel was prepared at 44 °C in oil bath with a dry N_2_ flow (30 mL/min), a strict stirring and flux for 24 h in a flask (250 mL). The resulting aminated SiO_2_ was repeatedly washed by ethanol to remove physically adsorbed APTES, and then dried at 50 °C in a vacuum (0.01 MPa) for 24 h. The aminated silica gel is referred as SiO_2_-APTES.

#### 3.1.2. Covalent Anchoring of Co-Schiff Bases on Aminated SiO_2_

Surface-aminated silica gel (SiO_2_-APTES) was covalently anchored by salicylaldehyde (SA), o-hydroxyacetophenone (HAP), 4-(diethylamino) salicylaldehyde (EASA) and 5-nitro salicylaldehyde (NSA) in the presence of cobaltous ions. Typically, the aminated SiO_2_ (6.4 g), 4-(diethylamino) salicylaldehyde (20 mmol), CH_2_Cl_2_ (100 mL) and Co(acac)_2_ (1.28 mmol, Sinopharm) were introduced into a flask (250 mL) at the same time. The covalent anchoring of the ligand EASA accompanied with the exchange for cobaltous ions via Co(acac)_2_ proceeded at 44 °C with a flowing N_2_ (30 mL/min) and a strict stirring/flux for 24 h. The obtained sample was repeatedly washed using CH_2_Cl_2_ followed by a drying at 50 °C in a vacuum (0.01 MPa). The catalyst synthesized by a so-called “one step” method is referred to as SiO_2_-APTES-EASA-Co, and the synthetic route of the catalyst and other catalysts with different Schiff-base ligands are illustrated in Scheme 1 and Scheme S1, respectively. Accordingly, the Co-Schiff base catalysts immobilized by the ligands SA, HAP and NSA are named as SiO_2_-APTES-SA-Co, SiO_2_-APTES-HAP-Co and SiO_2_-APTES-NSA-Co, respectively.

### 3.2. Characterization and Analysis

The fresh, spent catalysts and their intermediates were characterized, and the details are included in the Supplementary Materials. Briefly, the elemental compositions of the synthesized catalysts were determined via an element analyzer (Vario EL Cube, Elementar, Germany) for carbon and nitrogen elements and an inductively coupled plasma optical emission spectrometer (ICP-OES, Optima 7300DV, PerkinElmer, Waltham, MA, USA) for the cobalt element. The textural properties of the catalysts were analyzed by low temperature N_2_ adsorption–desorption carried out at 77 K on a ASAP 2010 micropore analysis system (Micromeritics, Norcross, GA, USA). The morphology of the catalysts and the dispersion of the elements in them were investigated by a Tecnai G2 F30 S-TWIN high-resolution electron microscope (Philips, Amsterdam, The Netherlands). FT-IR spectra of the catalysts were recorded to ascertain their structure using a self-supporting wafer diluted by KBr with a concentration of ca. 1% on an FT-IR spectrometer (TENSOR 27, Bruker, Karlsruhe, Germany). The UV-Vis spectra were recorded by diffuse reflectance on a UV-Vis (Cintra1010, East & West Analytical Instruments, Inc., Beijing, China) spectrometer equipped with a diffuse reflectance attachment using BaSO_4_ as a reference. The ^13^C NMR spectra of the catalysts were acquired at room temperature on a solid NMR spectrometer (Avance III, 400 WB, Bruker, Karlsruhe, Germany) to investigate the organic moiety anchored on SiO_2_, and the assignment of carbons was performed by means of the tool MestReNova. The thermal stability of the catalysts was analyzed on a thermal gravimetric analyzer (Pyris 1 TGA, PerkinElmer, Shelton, CT, USA) in the temperature range of 30–800 °C at a temperature ramp of 10 °C/min under atmospheric N_2_. XP spectra of the catalysts were obtained on an ESCALAB electron spectrometer to investigate chemical states of the elements.

### 3.3. Catalytic Tests

#### 3.3.1. Catalytic Activity

The catalytic performance of the immobilized Co-Schiff bases was evaluated via selective oxidation of toluene to benzaldehyde under solvent-free conditions. The tests were performed in a PTFE-lined autoclave (50 mL), and reaction temperatures were monitored by a steel-armored thermocouple inserted in the autoclave coaxially. Typically, toluene (10 g), Chlorobenzene (2.0 g, internal standard), NHPI (toluene/NHPI = 40, molar) and Co-Schiff base catalyst (NHPI/Co = 80, molar) were added to the autoclave followed by a repeated purging by O_2_ (99.999%) before tests. The catalytic tests were usually carried out at 100 °C for 1 h in oxygen (2.6 MPa). The resulting reaction mixture was separated by centrifugation to recycle the anchored Co-Schiff base catalysts, and the solution obtained was analyzed by a gas chromatographer (GC-2010, Shimadzu, Kyoto, Japan) equipped with a capillary column (SGE AC-10, 30 m × 0.22 mm) and a flame ionization detector (FID). Every catalytic test was repeated at least 3 times. Toluene conversion and the selectivities to benzaldehyde, benzyl alcohol, benzoic acid and dibenzyl ether were calculated via Equations (S1)–(S3) in the Supplementary Materials. The identification of the reaction products was determined by GC-MS (Trace ISQ, ThermoFisher, Waltham, MA, USA) equipped with a capillary column (DB-5, length: 30 m, i. d.: 0.25 mm, stationary phase thickness: 0.25 μm).

#### 3.3.2. Reusability

The recycled catalysts were separated by centrifugation and washed by ethanol 5 times to remove possible carbonaceous deposits, then dried in static air at 50 °C for 12 h. Typically, the spent catalyst was recorded as SiO_2_-APTES-EASA-Co-Ry, in which “y” denotes the times for which the catalyst was recycled. The spent catalysts were evaluated by the same method as the fresh ones.

## 4. Conclusions

Immobilized Co-Schiff base catalysts were successfully synthesized by amination pretreatment using commercial silica gel and APTES as the support and the linker, respectively, followed by a covalent anchoring and an ion exchange sequentially using salicylaldehyde (or its analogues) and the cobaltous acetyl acetone, respectively. The compositions, structural and textural properties, thermal stability and surface chemical states of the synthesized catalysts were well confirmed, exhibiting reliable anchoring of the ligands with a grafting density of 0.14 mmol/g. The catalysts demonstrated an excellent catalytic activity and reusability in the transformation from toluene to oxyfunctionalization products in the presence of NHPI under solvent-free conditions using dioxygen as the oxidant, and the effect of the ligands on their catalytic performance can be related to their electron-donating or -withdrawing properties.

## Figures and Tables

**Table 1:** Content of C, N and Co element in the fresh and the spent SiO_2_-APTES-EASA-Co catalysts.

Catalyst	C ^a^ (mmol/g)	N ^a^ (mmol/g)	Co ^b^ (mmol/g)	C/N ^c^ (Atomic)	Co/N ^c^ (Atomic)
SiO_2_-APTES-SA-Co	7.4	0.90	0.16	8.2 (12)	0.18 (0.5)
SiO_2_-APTES-HAP-Co	6.6	0.88	0.14	7.5 (13)	0.16 (0.5)
SiO_2_-APTES-EASA-Co	7.5	0.84	0.14	8.9 (8)	0.17 (0.25)
SiO_2_-APTES-NSA-Co	7.0	1.43	0.15	4.9 (12)	0.10 (0.25
SiO_2_-APTES-EASA-Co-R6	6.3	0.70	0.12	9.0 (8)	0.17 (0.25)

^a^ Determined by an element analyzer (Vario EL Cube, Elementar). ^b^ Determined by ICP-OES. ^c^ The values in parentheses are calculated according to Scheme 1 and Scheme S1.

**Table 2:** Textural properties of the synthesized the fresh and spent SiO_2_-APTES-EASA-Co catalysts in comparison with the support SiO_2_.

Catalyst	S_BET_ (m^2^/g) ^a^	Pore Volume (cm^3^/g) ^b^	Average Pore Diameter (nm) ^c^
SiO_2_	418	0.62	10.9
SiO_2_-APTES-SA-Co	124	0.38	10.0
SiO_2_-APTES-HAP-Co	114	0.37	9.0
SiO_2_-APTES-EASA-Co	147	0.44	9.0
SiO_2_-APTES-NSA-Co	117	0.39	9.4
SiO_2_-APTES-EASA-Co-R6	172	0.46	10.1

^a^ Determined by the BET method. ^b^ BJH desorption cumulative volume of pore between 0.85 nm and 150 nm. ^c^ BJH desorption average pore diameter.

**Table 3:** Catalytic performance of Co-Schiff base catalysts synthesized by different ligands for aerobic oxidation of toluene under solvent-free conditions ^a^.

Catalyst	Conversion of Toluene (%)	Selectivity (%)				
		BAH	BAL	BAC	DBE	Others
SiO_2_-APTES-SA-Co	19.2	15.5	11.2	68.4	4.1	1.0
SiO_2_-APTES-HAP-Co	20.0	14.7	9.9	71.0	4.1	0.4
SiO_2_-APTES-EASA-Co	20.8	14.3	11.5	70.2	3.2	0.8
SiO_2_-APTES-NSA-Co	12.1	18.9	12.4	64.7	3.6	0.4
EASA-Co ^b^	10.5	32.2	15.4	49.5	2.0	0.9

^a^ Reaction conditions: toluene/NHPI (40), NHPI/Co (80), 2.6 MPa, 100 °C, 1 h. BAH: benzaldehyde, BAL: benzyl alcohol, BAC: benzoic acid, DBE: dibenzyl ether; ^b^ 4 h.

**Table 4:** Effect of toluene/NHPI on the catalytic oxidation of toluene by molecular oxygen ^a^.

Toluene/NHPI	Conversion (%)	Selectivity (%)				
		BAH	BAL	BAC	DBE	Others
10	37.5	7.7	7.2	80.0	4.9	0.6
20	31.3	8.1	7.9	73.1	10.7	0.2
30	26.4	11.6	9.1	73.2	5.3	0.9
40	20.3	13.5	11.5	70.9	4.0	0.1

^a^ Reaction conditions: NHPI/Co (80, SiO_2_-APTES-EASA-Co), 2.6 MPa, 100 °C, 1 h. BAH: benzaldehyde, BAL: benzyl alcohol, BAC: benzoic acid, DBE: dibenzyl ether.

**Table 5:** Performance of the catalysts for solvent-free oxidation of toluene via molecular oxygen ^a^.

Catalyst	Conv. (%)	Selectivity (%)				
		BAH	BAL	BAC	DBE	Others
None	0	-	-	-	-	-
SiO_2_-APTES-EASA-Co ^c^	0	-	-	-	-	-
NHPI ^b^	0.8	74.6	25.4	0	0	0
NHPI, SiO_2_-APTES-EA SA-Co ^b^	31.3	8.1	7.9	73.1	10.7	0.2

^a^ Reaction conditions: toluene (10 g), 2.6 MPa, 100 °C, 1 h. BAH: benzaldehyde, BAL: benzyl alcohol, BAC: benzoic acid, DBE: dibenzyl ether. ^b^ Toluene/NHPI (20). ^c^ Toluene/Co (1600).

## Data Availability

Data supporting the results can be obtained by contacting the corresponding author.

## Supplementary Materials

The following supporting information can be downloaded at: https://www.mdpi.com/article/10.3390/molecules27165302/s1, Scheme S1: Synthetic illustration of covalently anchored cobaltous Schiff base catalysts on SiO_2_; Figure S1: TEM images of the covalently anchored Co-Schiff base catalysts. A, SiO_2_-APTES-SA-Co; B, SiO_2_-APTES-SA-Co; C, SiO_2_-APTES-SA-Co; D, SiO_2_-APTES-NSA-Co; Figure S2: The energy dispersive X-ray spectra of the fresh catalyst SiO_2_-APTES-EASA-Co; Figure S3: ^13^C NMR spectrum of SiO_2_-APTES-SA-Co; Figure S4: ^13^C NMR spectrum of SiO_2_-APTES-HAP-Co; Figure S5: ^13^C NMR spectrum of SiO_2_-APTES-NSA-Co; Figure S6: TGA curves of the covalently anchored Co-Schiff base catalysts; Figure S7: The survey XP spectra of the fresh and retrieved SiO_2_-APTES-EASA-Co catalysts; Table S1: Chemical shifts of carbon atoms in the fresh catalyst SiO_2_-APTES-EASA-Co and the catalyst SiO_2_-APTES-EASA-Co retrieved from the 6th catalytic test determined by ^13^C NMR spectra; Table S2: A comparison of the activity of the catalyst(s) for solvent-free oxidation of toluene compared with those reported in literatures in the past 5 years; Table S3: Effect of reaction time on the catalytic oxidation of toluene by molecular oxygen; Table S4: Performance of the catalyst SiO_2_-APTES-EASA-Co for solvent-free oxidation of cumene via molecular oxygen in presence of NHPI; Table S5: Performance of the catalyst SiO_2_-APTES-EASA-Co for solvent-free oxidation of ethylbenzene via molecular oxygen in presence of NHPI; Table S6: Performance of the catalyst SiO_2_-APTES-EASA-Co for solvent-free oxidation of ethylbenzene via molecular oxygen in presence of NHPI.

## Sample Availability

Some of the powder samples are available, but only in very small amounts.
