# Supplementary material for: Oxyfunctionalization of Benzylic C-H Bonds of Toluene Mediated by Covalently Anchored Co-Schiff Bases
Source: Molecules. 2022 Aug 19;27(16):5302. doi: 10.3390/molecules27165302 (PMC9416660; doi:10.3390/molecules27165302)
Supplement: Supplementary file 1 [file molecules-27-05302-s001.zip › molecules-1860664-supplementary.pdf]

Supplementary Materials

Oxyfunctionalization of Benzylic C–H Bonds of Toluene Mediated by Covalently Anchored Co-Schiff Bases

Guojun Shi *, Yuxin Liang, Hongyu Zhou, Zhengliang Zhao and Wenjie Yang

School of Chemistry and Chemical Engineering, Yangzhou University, Yangzhou 225002, China

Corresponding author:

Permanent address: School of Chemistry and Chemical Engineering, Yangzhou University, Yangzhou 225002, Jiangsu, China

Tel. and fax: +86-514-8793-7661

E-mail: gjshi@yzu.edu.cn

1. Experimental section

1.1. Synthesis of catalysts

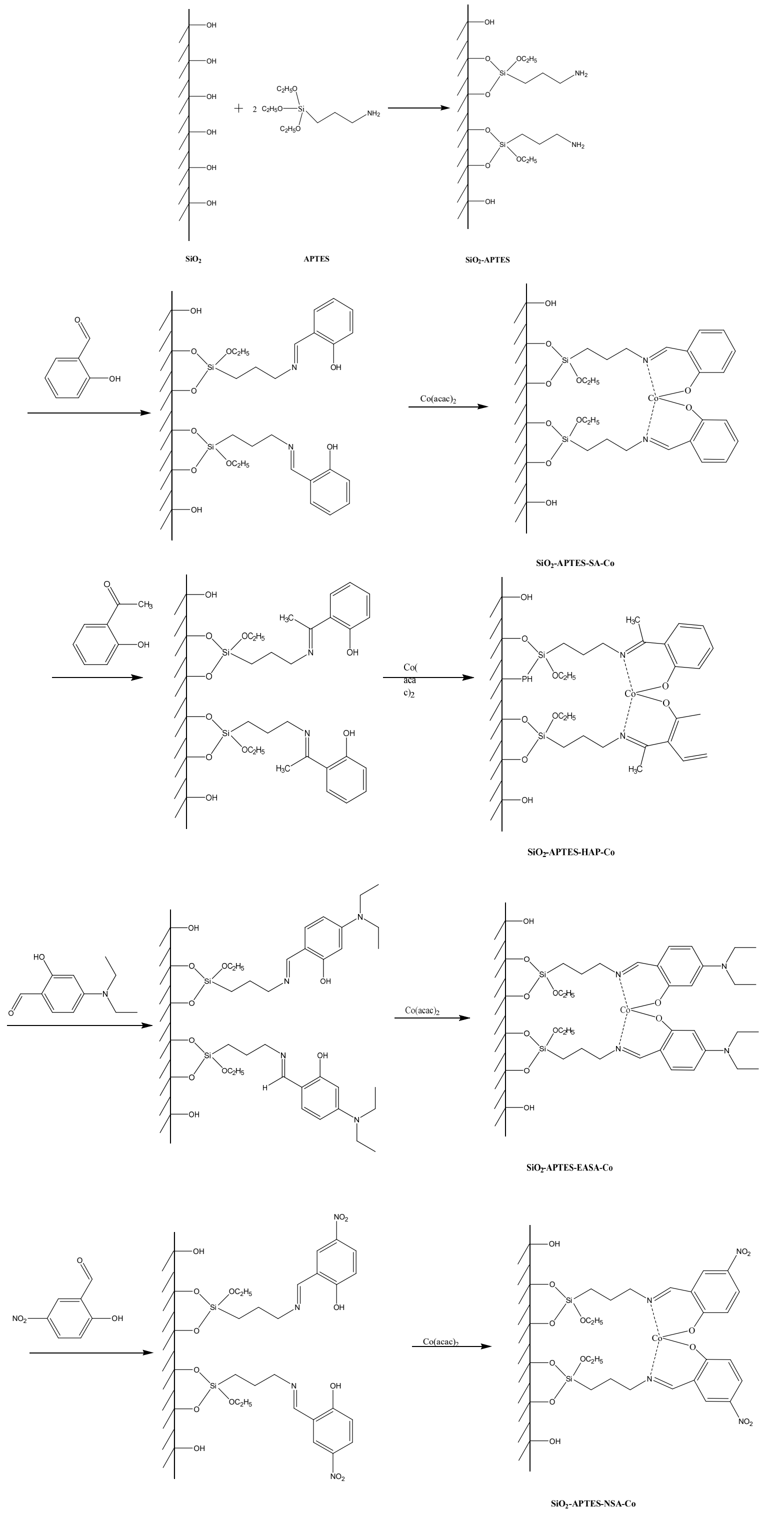

Scheme S1. Synthetic illustration of covalently anchored cobaltous Schiff base catalysts on SiO_2.

1.2. Characterization of catalysts

The contents of C and N elements were determined by a Vario EL Cube element analyzer. The samples with an accurate mass was burned using oxygen (99.999) in the presence of CuO as a catalyst, the resulting gaseous mixture was analyzed by a gas chromatographer and calculated to determine the concentration of C, H and N elements in the synthesized catalysts.

The actual Co loadings in the synthesized catalysts was analyzed an inductively coupled plasma optical emission spectrometer (ICP-OES, PerkinElmer, Optima 7300DV). The accurate amount of samples was carefully grinded and dissolved in a solution of hydrofluoric acid, and then diluted roughly to 5 ppm before analysis.

Low temperature N_2 adsorption–desorption was operated at 77 K on a Micromeritics ASAP 2010 micropore analysis system. About 50 mg of the sample was degassed in vacuum at 393 K for 2 h before adsorption. The BET equation was used to determine the specific surface areas of the samples, and the BJH mode to calculate the pore volume and average pore diameter.

TEM images were obtained using a Tecnai 12 electron microscope to determine the morphologies of the catalysts. The samples were dispersed in deionized water by the means of ultrasonic bath for 15 minutes after a careful grinding, and then drop-casted onto a carbon-coated copper grid. The HRTEM and element mapping were carried out by a Tecnai G2 F30 S-TWIN electron microscope.

FT-IR spectra were recorded on a TENSOR 27 spectrometer with a resolution of 4 cm^{-1} and a scanning scope of 400~4000 cm^{-1}. The self-supporting wafer diluted by KBr was prepared with a sample concentration of 1%.

Thermogravimetric analyses were carried out on a thermal gravimetric analyzer (Pyris 1 TGA, PerkinElmer) to investigate the grafting concentration and the thermal stability of the prepared SiO_2-bound NHPI catalyst. A temperature range 30–1000 ^{o}C and a scan rate of 10 ^{o}C/min were adopted under a N_2 atmosphere.

The UV-Vis spectra were recorded by diffuse reflectance on a Cintra10e UV-Vis spectrometer equipped with a diffuse reflectance attachment. $BaSO_4$ was used as a reference. All spectra were recorded at room temperature under ambient atmosphere. A sample holder was used to support the wafer of a sample with about 100 mg.

XPS measurements were performed on an ESCALAB electron spectrometer. Monochromatic Al *Kα* (1486.6 eV, 15 kV) was used as incident radiation. The obtained binding energies were determined with an overall resolution better than 0.05 eV. The C 1s binding energy from adventitious hydrocarbon was applied as a charge reference and fixed at 284.6 eV.

^{13}C NMR spectra of the catalysts were measured at room temperature on a solid NMR spectrometer (Bruker, Avance III, 400 WHz). The catalysts were dried and carefully ground before analysis, and then filled in a tube for tests.

1.3. Catalytic test and analysis

Toluene conversion is calculated by Eq. S1.

Eq. S1

$$Toluene\ conversion = \frac{Converted\ toluene}{Total\ toluene\ in\ raw\ mateial} \times 100\%$$

The selectivities to benzaldehyde, benzyl alcohol and benzoic acid were calculated via according to Eq. 2.

Eq. S2

$$Selectivity\ to\ benaldehyde = \frac{Toluene\ converted\ into\ benzaldehyde}{Total\ converted\ toluene} \times 100\%$$

The selectivities to benzyl alcohol and benzoic acid can be calculated by a similar method as Fig. S2. However, the selectivity to dibenzyl ether calculated by Eq. 3S because dibenzyl ether is formed by a condensation of benzyl alcohol (generated from toluene).

Eq. S3

$$Selectivity\ to\ dibenzyl\ ether = \frac{Toluene\ converted\ into\ dibenzyl\ ether}{Total\ converted\ toluene} \times 2 \times 100\%$$

2. Properties of catalysts

Table S1. Chemical Shifts of carbon atoms in the fresh catalyst SiO_2-APTES-EASA-Co and the catalyst SiO_2-APTES-EASA-Co retrieved from the 6th catalytic test determined by ^{13}C NMR spectra.

Chemical shift (ppm)	Numbers of carbon atom(s) [a]
10.2	1, 2, 3, 4, 27, 28
17.4	31, 32
22.5	25, 26
44.4	5, 6, 7, 8
53.9	23, 24
58.7	29, 30
99.7	13, 14
108.1	11, 12
124.8	19, 20
135.6	15, 16
155.9	9, 10
164.9	17, 18
177.2	21, 22

[a] The same as those shown in Figure 4.

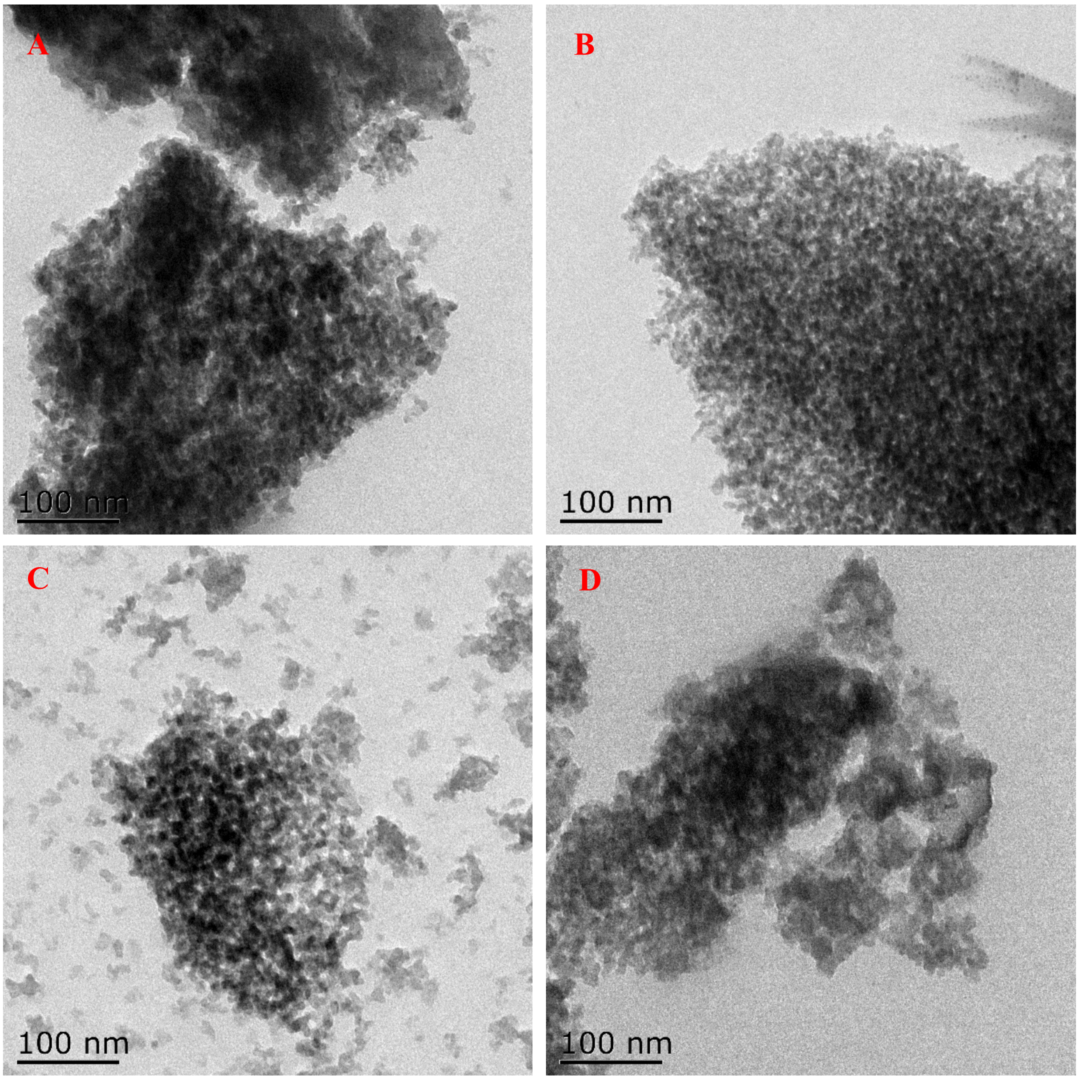

Figure S1. TEM images of the covalently anchored Co-Schiff base catalysts. A, SiO_2-APTES-SA-Co; B, SiO_2-APTES-SA-Co; C, SiO_2-APTES-SA-Co; D, SiO_2-APTES-NSA-Co.

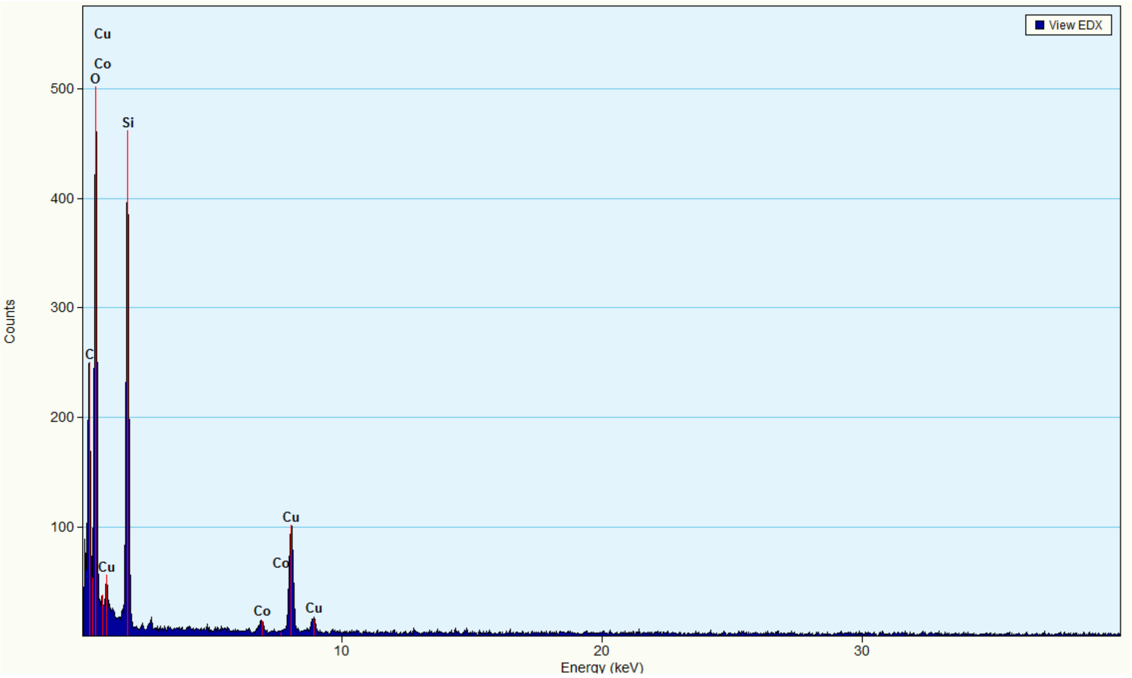

Figure S2. The energy dispersive X-ray spectra of the fresh catalyst SiO_2-APTES-EASA-Co.

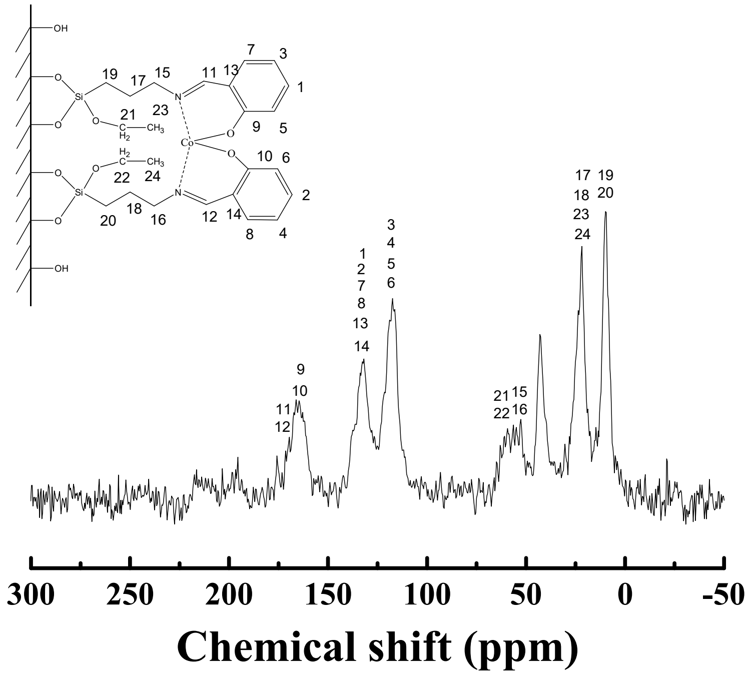

Figure S3. ^{13}C NMR spectrum of SiO_2-APTES-SA-Co.

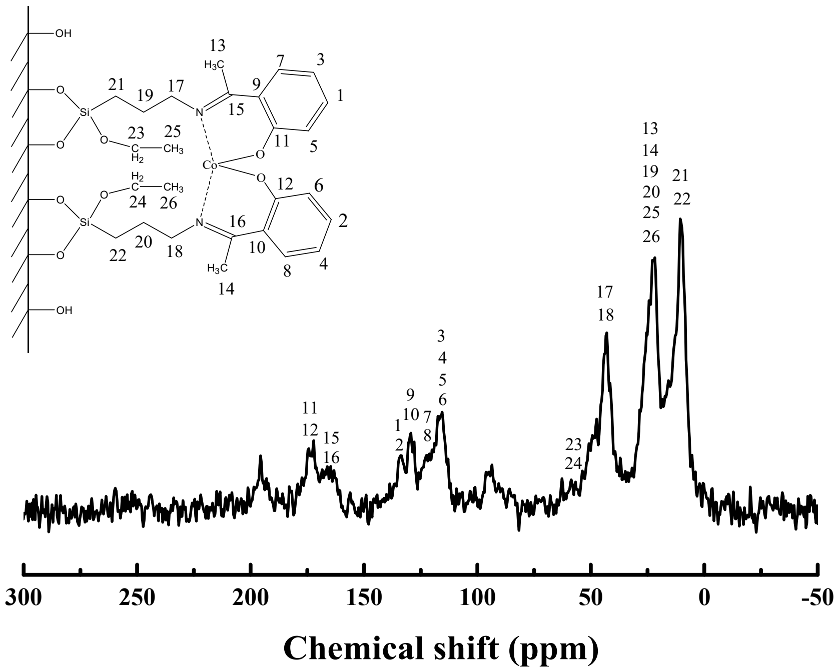

Figure S4. ^{13}C NMR spectrum of SiO_2-APTES-HAP-Co.

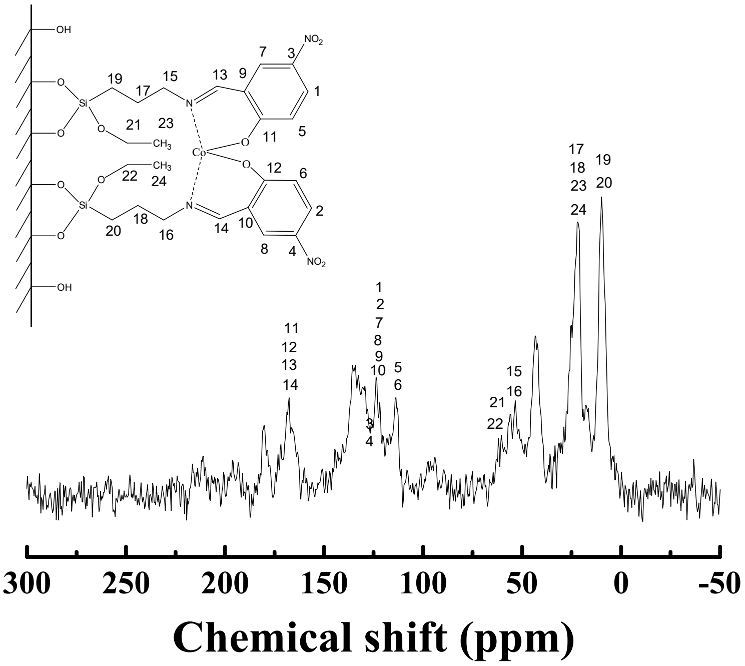

Figure S5. ^{13}C NMR spectrum of SiO_2-APTES-NSA-Co.

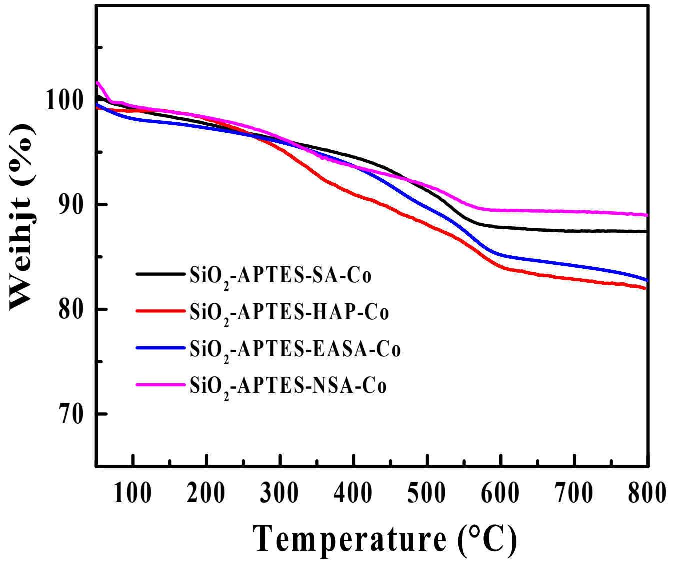

Figure S6. TGA curves of the covalently anchored Co-Schiff base catalysts.

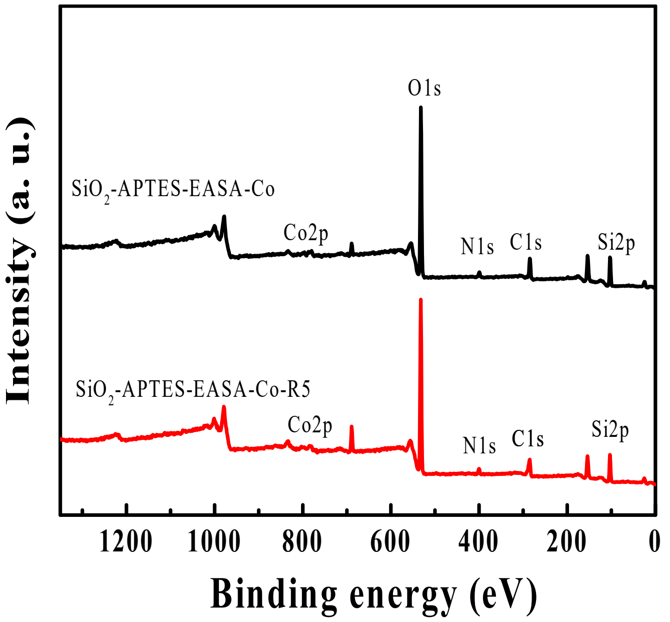

Figure S7. The survey XP spectra of the fresh and retrieved SiO_2-APTES-EASA-Co catalysts.

3. Catalytic performance

Table S2. A comparison of the activity of the catalyst(s) for solvent-free oxidation of toluene compared with those reported in literatures in the past 5 years.

Entry	Catalyst(s)	Reaction conditions	Oxidant	Toluene conversion (%)	Reference
1	Ag/SBA-15	120 °C, 1 atm	H_2O_2	39	Catal. Lett. (in press) doi.org/10.1007/s10562-021-03845-9
2	Fe-Bi_2WO_6	RT, 1 atm	O_2	1.35	Ind. Eng. Chem. Res. (in press), 10.1021/acs.iecr.0c02872
3	Cu/MCM-22	90 °C, 1 atm	H_2O_2	9	New J. Chem. 2019 (43) 4406
4	Ag/SiO_2	110 °C,	O_2	85	ACS Sustainable Chem. Eng. 8 (2020) 5856
5	Co-Schiff base, NHPI	100 °C, 2.6 MPa	O_2	37.5	In this work

Table S3. Effect of reaction time on the catalytic oxidation of toluene by molecular oxygen.

Time (h)	Conv. (%)	Selectivity (%)				
		BAH	BAL	BAC	DBE	Others
1/60	7.9	36.5	19.0	39.8	4.4	0.2
1/6	9.2	32.0	17.3	44.4	5.5	0.8
0.5	14.2	22.6	12.9	58.9	5.1	0.5
1	20.8	14.3	11.5	70.2	3.2	0.8

Reaction conditions: Catalyst (SiO_2-APTES-EASA-Co), Toluene/NHPI (40), NHPI/Co (80), 2.6 MPa, 100 °C, 1 h. BAH: Benzaldehyde, BAL: Benzyl alcohol, BAC: Benzoic acid, DBE: Dibenzyl ether.

Table S4. Performance of the catalyst SiO_2-APTES-EASA-Co for solvent-free oxidation of cumene via molecular oxygen in presence of NHPI.

Substrate	Conv. (%)	Selectivity (%)				
		CHP	AP	AC	PP	AMS
Cumene	37.8	73.2	9.8	1.3	13.2	2.4

Reaction conditions: Cumene/NHPI (40), NHPI/Co (80), 2.6 MPa, 100°C, 1 h. CHP: Cumene hydroperoxide, AP: Acetophenone, AC: Acetone, PP: 2-phenyl-2-propanol, AMS: α-Methyl styrene.

Table S5. Performance of the catalyst SiO_2-APTES-EASA-Co for solvent-free oxidation of ethylbenzene via molecular oxygen in presence of NHPI.

Substrate	Conv. (%)	Selectivity (%)			
		BAH	AP	1-PEO	Others
Ethylbenzene	26.1	0.9	72.7	19.8	6.6

Reaction conditions: Ethylbenzene/NHPI (40), NHPI/Co (80), 2.6 MPa, 100°C, 1 h. BAH: Benzaldehyde, AP: Acetophenone, 1-PEO: 1-Phenylethanol.

Table S6. Performance of the catalyst SiO_2-APTES-EASA-Co for solvent-free oxidation of ethylbenzene via molecular oxygen in presence of NHPI.

Substrate	Conv. (%)	Selectivity (%)			
		p-MBAH	p-MBAL	p-MBAC	Others
Paraxylene	57.8	20.6	9.4	66.3	3.7

Reaction conditions: Paraxylene/NHPI (40), NHPI/Co (80), 2.6 MPa, 100°C, 1 h. p-MBAH: p-Methylbenzaldehyde, p-MBAL: p-Methyl benzyl alcohol, p-MBAC: p-Methyl benzoic acid, Others: p-Methylbenzyl acetate and p-methylbenzyl formate.
